# Extradural contralateral S1 nerve root transfer for spastic lower limb paralysis

**DOI:** 10.7555/JBR.37.20230068

**Published:** 2023-09-28

**Authors:** Jiang Cao, Jie Chang, Chaoqin Wu, Sheng Zhang, Binyu Wang, Kaixiang Yang, Xiaojian Cao, Tao Sui

**Affiliations:** 1 Department of Orthopedics, the First Affiliated Hospital of Nanjing Medical University, Nanjing, Jiangsu 210029, China; 2 Department of Orthopedics, the Second Affiliated Hospital of Nanjing Medical University, Nanjing, Jiangsu 210029, China

**Keywords:** paralysis, spinal nerve roots, nerve transfer, ankle joint

## Abstract

The current study aims to ascertain the anatomical feasibility of transferring the contralateral S1 ventral root (VR) to the ipsilateral L5 VR for treating unilateral spastic lower limb paralysis. Six formalin-fixed (three males and three females) cadavers were used. The VR of the contralateral S1 was transferred to the VR of the ipsilateral L5. The sural nerve was selected as a bridge between the donor and recipient nerve. The number of axons, the cross-sectional areas and the pertinent distances between the donor and recipient nerves were measured. The extradural S1 VR and L5 VR could be separated based on anatomical markers of the dorsal root ganglion. The gross distance between the S1 nerve root and L5 nerve root was 31.31 (± 3.23) mm in the six cadavers, while that on the diffusion tensor imaging was 47.51 (± 3.23) mm in 60 patients without spinal diseases, and both distances were seperately greater than that between the outlet of S1 from the spinal cord and the ganglion. The numbers of axons in the S1 VRs and L5 VRs were 13414.20 (± 2890.30) and 10613.20 (± 2135.58), respectively. The cross-sectional areas of the S1 VR and L5 VR were 1.68 (± 0.26) mm ^2^ and 1.08 (± 0.26) mm ^2^, respectively. In conclusion, transfer of the contralateral S1 VR to the ipsilateral L5 VR may be an anatomically feasible treatment option for unilateral spastic lower limb paralysis.

## Introduction

Injuries to the brain parenchyma from a stroke or a brain hemorrhage can result in spastic paralysis of the limbs. Although upper limb paralysis is more common than lower limb paralysis, the patients may have both, and a considerable number of patients also have lower limb paralysis only. Lower limb spastic paralysis is associated with foot ptosis, abnormal posture, increased muscle tension, and altered balance, all of which seriously impair the patients' daily living ability, disrupt productivity, and impose an economic burden on patients' families and society ^[ 1] ^. Lower limb spastic paralysis recovers more slowly than hemiplegia of the upper extremities. Some cases with a lower limb spastic paralysis are mainly concentrated in the distal extremities, including the lower legs and ankle joints ^[ 2] ^. It is estimated that approximately 60% of stroke survivors cannot walk normally due to lower limb spastic paralysis, especially due to ankle spasm ^[ 3] ^. Therefore, ankle spasm is a leading cause of disability in these patients ^[ 4] ^.

Existing treatment methods for hemiplegia of the lower limbs include drug therapy, electrical stimulation, underwater training, and lower limb robotic rehabilitation; however, these methods cannot achieve ideal results, because the main impediments to walking are lower limb spasm and ankle weakness.

Recently, a few investigators have begun focusing on peripheral nerve transplantation for the treatment of limb paralysis and bladder dysfunction caused by central nerve injury ^[ 5– 6] ^. Xiao *et al* ^[ 7]^ treated the neurogenic bladder by anastomosing the S2 and S3 nerve roots with the L5 or S1 nerve roots. Nagano *et al* ^[ 8]^ used the intercostal nerve transplantation to treat brachial plexus injury. Zheng *et al* ^[ 9]^ treated spastic upper limb palsy by grafting the C7 nerve from the nonparalyzed side to the spastic paralyzed side of the upper limb. Yu *et al* ^[ 10]^ used contralateral hemi-5th-lumbar nerve transfer to treat incomplete spinal cord injury patients with unilateral lower limb dysfunction. These studies demonstrate that considerable progress has been achieved in using peripheral nerve transplantation for the treatment of limb dysfunction caused by central nervous system injury. Nevertheless, peripheral nerve transplantation has not yet been reported for the treatment of spastic lower limb paralysis secondary to chronic cerebral injury.

In our previous studies, we demonstrated the feasibility of epidural nerve root transfer for the treatment of spastic upper limb paralysis, lower limb paralysis, and neurogenic bladder after spinal cord injury ^[ 11– 14] ^. Among these, the incorporation of epidural nerve root transfer into the treatment of neurogenic bladder has yielded satisfactory results in clinical practice. The epidural nerve root transfer technique allows accurate separation of the anterior and posterior roots in the epidural space and ensures accurate anastomosis with the target nerve roots, thereby substantially increasing the efficiency of nerve regeneration and reducing the incidence of surgical complications.

Damage to the cerebral hemispheres due to stroke, cerebral hemorrhage, and other causes can cause unilateral spastic paralysis of the lower limbs. Meanwhile, ankle spasm and ankle weakness are important factors that impair walking. In the current study, we attempted to anastomose the contralateral S1 ventral root (VR) to the ipsilateral L5 VR to relieve ankle spasm and restore lower limb motor function.

## Material and methods

### Anatomical description

In the current study, six formalin-fixed cadavers (three males and three females) were selected from the Department of Anatomy at Nanjing Medical University. Their age ranged from 35 to 79 years, with the mean age of 63 years. Each of the donors signed a written informed consent before they died. The current study was approved by the Medical Ethical Committee of the First Affiliated Hospital of Nanjing Medical University (Approval No. 2022-SR-316). Those with a history of spinal surgery, spinal malformation, disc disease, and spinal fracture were excluded.

### Surgical procedure

**Figure 1:**
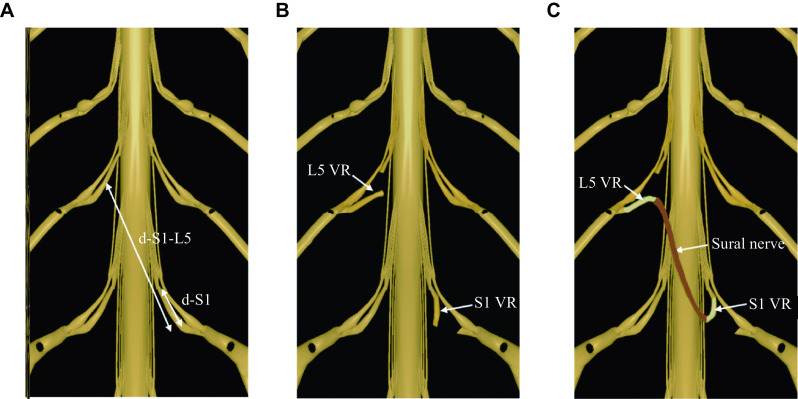
Schematic showing the relevant distances and nerve transfer alluded to in the study.

The cadavers were placed in the prone position. A posterior median approach incision was made to gradually dissect the paraspinal muscles and expose the bilateral articular processes and vertebral lamina. Next, laminectomy was performed, with the L5–S1 lamina and spinous process removed up to the facet joints on both sides. Bilateral L5 and S1 nerve roots were exposed and the VRs and the dorsal roots (DRs) were carefully separated in the epidural space. Ipsilateral L5 VRs were severed at the nerve root outlet, and contralateral S1 VRs were severed at the junction of VR and DR. Finally, the VR of ipsilateral L5 and contralateral S1 were anastomosed in the epidural space using 6-0 prolene suture (Ethicon, Cat. #8521P38, Cincinnati, Ohio, USA) with a sural nerve bridge. The schematic diagram of nerve transplantation is shown in ***Fig. 1***.

To assess whether the distance between the donor and recipient nerves was sufficient for tension-free anastomosis, we measured the length of the associated epidural nerve roots. After the operation, the nerve roots at both ends of the anastomosis were cut and examined under hematoxylin and eosin staining. The morphology, number of nerve fibers, and cross-sectional area of the specimens were assessed. To further confirm whether the distance of nerve transfer in this technique was sufficient, 3D reconstruction and data measurement were performed by diffusion tensor imaging (DTI) of patients without spinal disease (30 males and 30 females). The distance between L5 and S1 was measured.

### Statistical analysis

SPSS 26.0 (IBM Corp., Armonk, NY, USA) was used for statistical analysis. The normally distributed variables were expressed as mean ± standard deviation. Statistical analysis was performed using the unpaired Student's *t*-test. *P* < 0.05 was considered significant.

## Results

### Extradural anastomosis of the contralateral S1 VR to ipsilateral L5 VR

**Figure 2:**
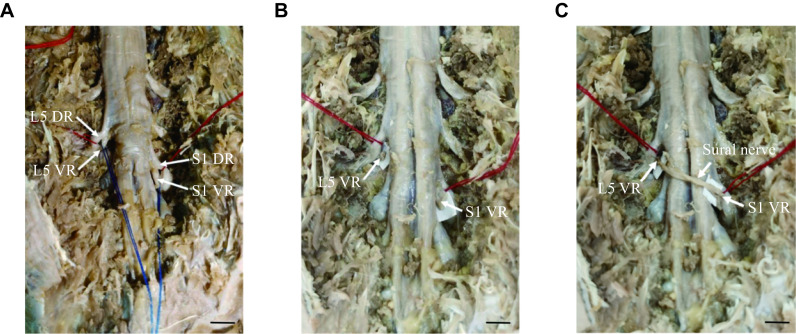
Extradural nerve anastomosis between the S1 VR and L5 VR in formalin-fixed cadaver.

The cadavers were placed in the prone position. Paraspinal muscles and soft tissue were dissected through a posterior median approach to expose the L5–S1 spinous process, bilateral vertebral plates, and articular processes in sequence. Then, a restricted laminectomy was performed to expose the dura mater and the L5, S1 epidural nerve root. The VRs and DRs were carefully separated at the DR ganglion location. After the epidural nerve roots were separated, the distal end of the contralateral S1 VR and the proximal end of the L5 VR near the DR ganglion were severed, and the distal end of the S1 VR was transferred to the proximal end of the L5 VR. We found that the S1 VR did not have sufficient length to be grafted to the L5 VR. Therefore, we excised the sural nerve of sufficient length as a bridge for nerve transplantation, as shown in ***Fig. 1***. The surgical procedure on the fixed body is shown in ***Fig. 2***.

### Nerve axon number and cross-sectional area of S1 VR, L5 VR, and sural nerves

**Figure 3:**
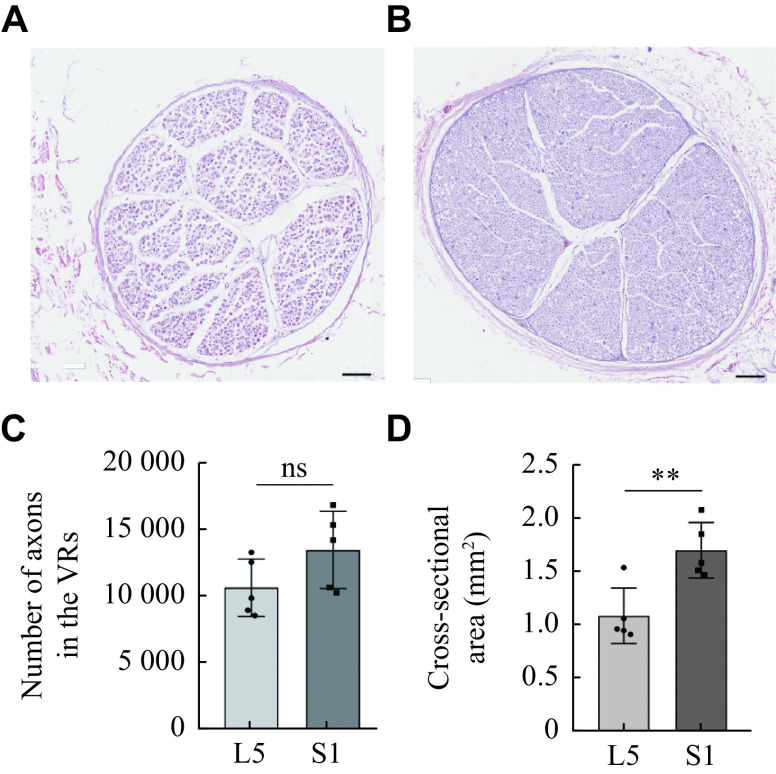
Hematoxylin and eosin staining of the S1 VR and L5 VR and the quantification of nerve axon number and cross-section area.

Cross sections of the S1 VR and L5 VR are shown in ***Fig. 3A*** and ***3B***. The S1 VR and L5 VR were present in one bundle, and the sural nerve was present in two bundles ^[ 15] ^. The numbers of axons in the S1 VRs and L5 VRs were 13414.20 (± 2890.30) and 10613.20 (± 2135.58), respectively ( ***Fig. 3C***). The cross-sectional areas of the S1 VR and L5 VR were 1.68 (± 0.26) mm ^2^ and 1.08 (± 0.26) mm ^2^, respectively ( ***Fig. 3D***). There was no significant difference in axon number between S1 VR and L5 VR, but the cross-sectional area of S1 VR was larger than that of L5 VR, which satisfied the condition of nerve regeneration ^[ 15] ^.

### The pertinent distance between the contralateral S1 VR and the ipsilateral L5 VR

**Figure 4:**
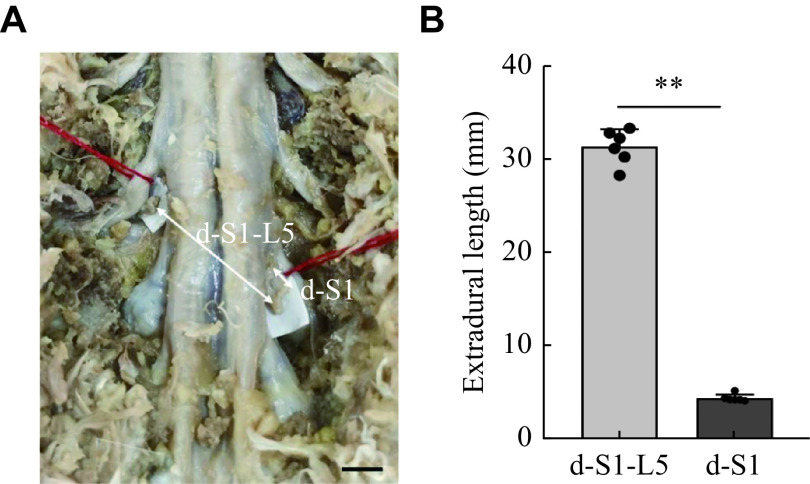
Extradural length of d-S1 and d-S1-L5.

**Figure 5:**
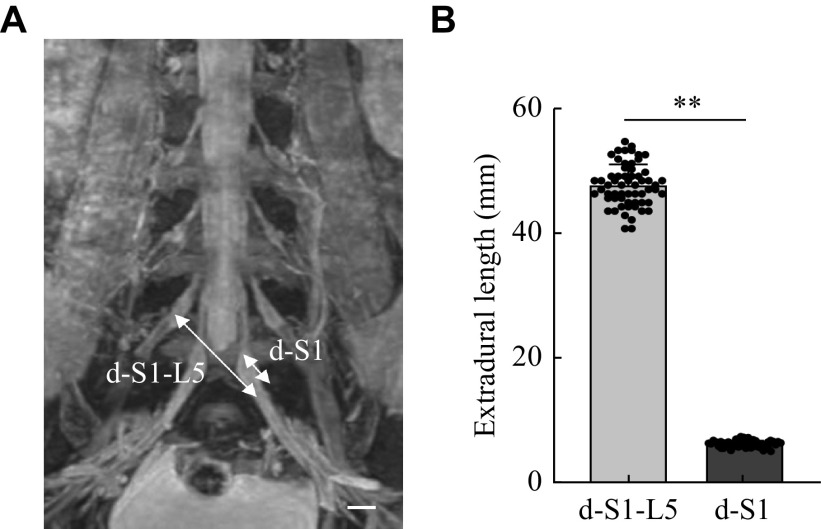
Extradural length of d-S1-L5 on DTI.

The distances between the contralateral S1 VR and the ipsilateral L5 VR were measured. In all six cases, the distance between the S1 VR and L5 VR was 31.31 (± 1.87) mm, and the distance between the outlet of the S1 from the spinal cord and the ganglion was 4.36 (± 0.39) mm ( ***Fig. 4***). As shown in ***Fig. 5***, the distance between the S1 VR and the L5 VR on DTI of 60 patients was 47.51 (± 3.23) mm, and the distance between the outlet of S1 from the spinal cord and the ganglion was 6.51 (± 0.55) mm. In both measurements, the distance between the S1 VR and L5 VR was greater than that between the outlet of S1 from the spinal cord and the ganglion.

## Discussion

Our study preliminarily confirmed the anatomic feasibility of nerve transplantation in restoring ankle motor function in patients with hemiplegia. Unilateral spasmodic limb motor dysfunction caused by cerebral ischemia or hemorrhagic injury is associated with muscle weakness, an increased muscle tone, and a reduced balance ability ^[ 16– 18] ^, leading to reduced joint motion, joint contracture, and foot drop. The reduced activity of the ankle muscles makes walking difficult. One way of improving walking difficulties is to use an ankle orthosis that stabilizes the foot and ankle during the loading phase, and to raise the toes during walking. The elevation of the foot provided by ankle orthoses can significantly improve walking speed, walking length, and balance ^[ 19– 22] ^. However, the use of ankle orthotics is controversial. Ankle orthotics may improve walking function in the short term, but in the long term, they do not reduce ankle spasms and may even worsen muscle atrophy and impede the recovery of normal function ^[ 23] ^. Furthermore, ankle orthotics may cause soft tissue injury, joint pain, muscle pain, fractures, and blood pressure changes ^[ 24] ^. With ankle fusion surgery, the same disadvantages apply ^[ 25– 26] ^. Once our treatment is introduced into the clinic, ankle spasms will be alleviated immediately after the L5 nerve root is severed. Successful reconstruction of the transplanted nerve alleviates ankle spasm and strengthen ankle motion, especially dorsiflexion and plantar flexion of the ankle, which is critical for walking. Compared with ankle orthoses, our method can not only relieve spasms and improve walking ability in the short term, but also restore ankle joint motor function in the long term.

There are two reasons for choosing the contralateral S1 nerve root as the donor nerve. First, the anterior root diameter of the S1 nerve is large, and the number of nerve fibers is sufficient, which is conducive to the growth of nerve roots. Second, amputation of the anterior S1 root on the healthy side does not impair the motor function of the healthy limb. Yang and Zhou *et al* ^[ 12– 13]^ demonstrated that anastomosis of the C7 nerve roots could treat spastic upper limb palsy, and this method did not damage the motion and sensation of the healthy side of the upper limb, while restoring the function of the affected side. Severing the S1 nerve root has a similar effect. Xiao ^[ 27]^ created an artificial somatic-central nervous system-autonomic reflex to treat neurogenic bladder, and they selected the L5 nerve root as the donor nerve; however, as the L5 nerve no longer innervated the lower limbs, some patients suffered foot ptosis, which seriously impaired the motor function of the lower limbs. Therefore, Lin *et al* ^[ 28]^ improved this approach selecting the S1 nerve root as a donor nerve for innervation of the bladder; their results showed that the adverse effects of S1 nerve root dissection could be compensated for by other nerve roots, thereby confirming the safety of the S1 nerve root dissection for nerve transplantation.

In our previous study, we demonstrated that the extradural transfer of the S1 VRs to the S2 and the S3 VR for restoring bladder dysfunction was surgically feasible ^[ 14] ^. We have applied this method to the clinic with satisfactory results. Two patients with neurogenic bladder dysfunction underwent our surgical procedure in which the S1 VR was transferred to the S2 and S3 VRs; and on the day and 12 months postoperatively, the muscle strength of the S1-innervated muscle did not decrease significantly, and also this advantage has been demonstrated in autopsies, animal studies and clinical trials ^[ 14, 29– 30] ^. Transfer of the S1 to the S2/3 nerve roots has been used in the artificial somatic-CNS-autonomic reflex arc procedure, which has been widely performed worldwide ^[ 27] ^.

The L5 nerve is an important component of the sciatic nerve that innervates the ankle joint, toes, and calf muscles ^[ 31] ^. Reconstruction of the L5 nerve function using the contralateral S1 nerve graft can significantly restore the motor function of the ankle joint and toe. At the same time, due to the diverse functions of the sciatic nerve, this method can also restore the function of other lower limb muscles to a certain extent. Amputation of the anterior L5 nerve root provides immediate relief from ankle spasms; therefore, a certain degree of ankle function can be restored without waiting for nerve regeneration ^[ 32– 33] ^, thus, vastly improving the patient's confidence in recovery.

The main determinants of nerve regeneration after nerve transplantation include the distance between the ipsilateral and contralateral nerves and the number of donor nerve axons ^[ 34] ^. In the current study, we measured the distance between the S1 VR and L5 VR. The distance between the donor and recipient nerves was found to be too long, and the contralateral S1 VR was seemingly under tension when being transferred to the ipsilateral L5 VR. Tension anastomosis is not conducive to nerve regeneration; furthermore, the donor nerve compresses the spinal cord as it crosses the spinal cord ^[ 35] ^. Therefore, another nerve must be selected as a bridge between the donor and recipient nerve. We choose the sural nerve as the bridging nerve. The condition for nerve regeneration is that the number of axons in the donor nerve must be at least 40% of those in the recipient nerve ^[ 15] ^. The greater the number of axons and the larger cross-sectional area, the more efficient nerve regeneration and the better the clinical efficacy will be. The results of the current study suggest that the S1 VR and sural nerve may provide adequate axon numbers and cross-sectional area. The distance between the contralateral S1 VR and the ipsilateral L5 VR on DTI was longer than that in cadavers. The reason may be that when we dissect the cadaver, the nerve roots have a certain degree of extensibility and mobility. Based on this scenario, the length of the bridging nerve may be shorter than what is actually required. When we chose the sural nerve as the bridging nerve, the maximum length of the bridging nerve was measured on DTI, although the actual length used may be shorter than it.

Our previous studies have shown that epidural precision nerve root transplantation is more beneficial for nerve regeneration than other graft techniques, possibly being attributed to the following reasons. First, this method accurately transfers the motor nerve fibers of the donor nerve to the motor nerve fibers of the recipient nerve, avoiding nerve fiber mismatch and improving the efficiency of nerve regeneration ^[ 34, 36] ^. Second, the separation and nerve anastomosis in the epidural space reduces the risk of nerve root injury and cerebrospinal fluid leakage. Finally, because the epidural space encloses the nerve root, the epidural technique can enable strong anastomosis of the nerve and facilitate nerve regeneration. When the nerve regeneration is complete, the cortex can hopefully regain control of the affected limb.

Several limitations of the current study need to be addressed in the future. Compared with traditional non-invasive methods, this type of spinal surgery can predispose the patient to surgical complications, such as bleeding, infection, and the risk of being bedridden. As cadaveric specimens are difficult to obtain, the number of cadavers in the current study was insufficient, and additional samples are needed in the future to confirm our findings.

Taken together, our study demonstrates that extradural transfer of the contralateral S1 VR to the ipsilateral L5 VR is anatomically feasible as a treatment option for unilateral spastic paralysis caused by cerebral ischemia or hemorrhagic injury. These findings need to be validated further in clinical practice.
